# The burden of dengue, source reduction measures, and serotype patterns in Myanmar, 2011 to 2015–R2

**DOI:** 10.1186/s41182-017-0074-5

**Published:** 2017-11-02

**Authors:** Pwint Mon Oo, Khin Thet Wai, Anthony D. Harries, Hemant Deepak Shewade, Tin Oo, Aung Thi, Zaw Lin

**Affiliations:** 1 Central Vector Borne Disease Control Programme, Department of Public Health, Ministry of Health and Sport, Nay Pyi Taw, Myanmar; 2 Department of Medical Research, Yangon, Myanmar; 3 International Union against Tuberculosis and Lung Disease, Paris, France; 4 London School of Hygiene and Tropical Medicine, London, UK; 5 International Union against Tuberculosis and Lung Disease (The Union), South-East Asia Office, New Delhi, India

**Keywords:** Children, Dengue, Dengue hemorrhagic fever, Dengue case fatality, Dengue virus serotypes, Health education, Larval control measures, Myanmar, Space spraying

## Abstract

**Background:**

Myanmar is currently classified as a high burden dengue country in the Asian Pacific region. The Myanmar vector-borne diseases control (VBDC) program has collected data on dengue and source reduction measures since 1970, and there is a pressing need to collate, analyze, and interpret this information. The aim of this study was to describe the burden of hospital-based dengue disease, dengue control measures, and serotype patterns in Myanmar between 2011 and 2015.

**Methods:**

This was a cross-sectional study using annual records from the Dengue Fever/Dengue Hemorrhagic Fever Prevention and Control Project in Myanmar.

**Results:**

Between 2011 and 2015, there were a total of 89,832 cases and 393 deaths in hospitals, with 97% of cases being in children. In 2013 and 2015, there was an increased number of cases, respectively at 21,942 and 42,913, while during the other 3 years, numbers ranged from 4738 to 13,806. The distribution of dengue deaths each year mirrored the distribution of cases. Most cases (84%) occurred in the wet season and 54% occurred in the delta/lowlands. Case fatality rate (CFR) was highest in 2014 at 7 per 1000 dengue cases, while in the other years, it ranged from 3 to 5 per 1000 cases. High CFR per 1000 were also observed in infants < 1 year (CFR = 8), adults ≥ 15 years (CFR = 7), those with disease severity grade IV (CFR = 17), and those residing in hilly regions (CFR = 9). Implementation and coverage of dengue source reduction measures, including larval control, space spraying, and health education, all increased between 2012 and 2015, although there was low coverage of these interventions in households and schools and for water containers. In the 2013 outbreak, dengue virus serotype 1 predominated, while in the 2015 outbreak, serotypes 1, 2, and 4 were those mainly in circulation.

**Conclusion:**

Dengue is a serious public health disease burden in Myanmar. More attention is needed to improve monitoring, recording, and reporting of cases, deaths, and vector control activities, and more investment is needed for programmatic research.

## Background

Dengue fever is a mosquito-borne tropical disease that usually lasts 2 to 7 days and is caused by the dengue virus [1, 2]. In a small proportion of cases, the disease develops into (a) life-threatening dengue hemorrhagic fever (DHF) or (b) dengue shock syndrome (DSS). There are no specific drugs for the treatment of dengue, which is merely supportive. The infection is spread by two species of *Aedes* mosquito, *Aedes aegypti* (the principal vector) and *Aedes albopictus.* The virus has four distinct, but closely related, serotypes. Recovery from infection by one serotype provides lifelong immunity against that particular serotype. Cross-immunity to the other serotypes occurs, but this is only partial and temporary, and subsequent infections by other serotypes increase the risk of that person developing severe dengue complications such as DHF or DSS. A novel vaccine (Dengvaxia) has been developed and has been shown to be effective [3–5], but, although it is licensed and in use, it is not yet fully deployed. Thus, prevention is currently focused on reducing the mosquito habitats and limiting human exposure to mosquito bites.

The incidence of dengue has grown dramatically around the world in recent decades, with a 30-fold increase in estimated cases in the last 50 years. On the basis of population data and geostatistical models, the burden of dengue infections globally is estimated at 390 million infections per year, with 96 million of those presenting clinically with mild, moderate, or severe disease [6, 7]. Further modeling suggests that 3.9 billion people living in 128 countries are at risk of infection with dengue viruses [7]. In the World Health Organization (WHO) South-East Asia region and Western Pacific Region, some 1.8 billion people (more than 70% of the population) are at risk of dengue, and this poses a substantial economic and disease burden on these countries [1, 8].

To combat this growing problem in the region, the Asia Pacific Dengue Strategic Plan (2008–2015) was developed with the goal of reversing the rising trend of dengue cases by enhancing preparedness to detect, characterize, and contain outbreaks rapidly and to stop the spread of the virus to new areas [9]. Progress is being made with implementation combined with operational and surveillance response research [10].

Myanmar has seen an escalating number of cases of dengue in the last 10 years, despite vector control efforts, and is currently classified as a high burden dengue country in the Asian Pacific region [8]. There is a prevention and treatment program which works as follows. Patients with dengue are diagnosed in hospitals of all States/Regions of Myanmar, with the diagnosis mainly based on clinical criteria according to 2011 WHO guidelines [11]. Rapid diagnostic test kits for dengue are available, but these are in limited supply and are rarely used. Serotype confirmation can be carried out, but this requires polymerase chain reaction (PCR), which is available at the National Health Laboratory under the Department of Public Health and the Department of Medical Research. PCR is done mainly through research and surveillance in response to outbreaks, but each year, children with dengue are also asked to provide specimens so that information can be obtained about circulating serotypes [12, 13]. Once patients are notified with dengue, source reduction measures (mainly control of mosquito larvae and adult mosquitoes) are implemented along with health education and social mobilization [14].

The Myanmar vector-borne diseases control (VBDC) program has collected data on dengue cases, dengue deaths, and vector control measures since 1970, but there is a pressing need to collate, analyze, and interpret this information and, particularly, to generate location-specific spot maps of the burden of dengue in the country. For a resource-constrained country like Myanmar, this information will enhance cost-effective mitigation strategies and help to promote community engagement for dengue control. The aim of this study, therefore, was to describe the burden of dengue disease, dengue control measures, and serotype patterns in Myanmar over a 5-year period between 2011 and 2015. Specific objectives were to describe for each year: (i) public hospital-reported cases of dengue and deaths, stratified by age, gender, urban/rural residence, disease severity grade, season, and geographical ecology; (ii) dengue vector control measures, including mass larval control, space spraying (fogging), and health education; and (iii) serotype patterns of dengue virus from selected states/regions that were identified in the National Health Laboratory.

## Methods

### Study design

This was a cross-sectional descriptive study using annual records from the Dengue Fever/Dengue Hemorrhagic Fever (DF/DHF) Prevention and Control Project in Myanmar.

### Setting

#### General setting

Myanmar is located in the Southeast Asia Region, bordering the Republic of China on the north and northeast, Laos on the east, Thailand on the southeast, Bangladesh on the west, and India on the northwest. The country is divided administratively into the Nay Pyi Taw Council Territory and 14 states and regions and consists of 74 districts, 330 townships, 398 towns, 3065 wards, 13,619 village tracts, and 64,134 villages. The main geographical features of the country are the delta region and the central plain surrounded by hills and mountains. Myanmar enjoys a tropical climate with three distinct seasons: hot (February to April), wet (May to September), and cool (October to January) [15]. Myanmar has a population of 51 million people with an urban-rural population ratio of 30:70. It has an area of 0.6 million square kilometer and a population density of 76 per km^2^ [15].

#### Myanmar DF/DHF prevention and control project

In Myanmar, the DF/DHF surveillance program was launched in 1964 and dengue fever was made a notifiable disease in 1966. In 1968, the National Committee on DHF was formed and the *Aedes* Mosquito Control Unit was established. In 1969, sporadic cases of DHF were recorded in Yangon. The first epidemic of DHF in Myanmar was recorded in 1970. At that time, the disease was confined to the Yangon Division only. From 1974 onwards, DHF began to spread to other states and divisions and the disease is now endemic in all states and regions (divisions) except Chin State.

The current National DHF Control Strategy has followed the Asian Pacific Regional Dengue Strategic Plan (2008–2015) [9]. The major goal is to reduce the incidence rates of DF and DHF. The main components of the plan are (i) effective disease and vector surveillance systems based on reliable laboratory and health information; (ii) disease prevention through selective, stratified, and integrated vector control with community education and engagement; (iii) emergency preparedness to prevent and control outbreaks with appropriate contingency plans; (iv) prompt case management of DF/DHF including early recognition of signs and symptoms to prevent mortality; (v) increased awareness of the community about DF/DHF prevention, control, and management through information, education, and communication; and (vi) improved management and technical support systems and strengthened health facilities for health sector development.

##### Dengue case surveillance

Cases that are admitted to hospital and diagnosed with possible dengue [dengue fever (DF) and DHF/DSS] based on WHO criteria [11] are notified to the health department that then introduces dengue source reduction measures as described below. Case records are kept at the hospital and a focal person from the VBDC team collects these data on a weekly basis through fax, electronic mail, and phone calls. It is estimated that data are collected from 90% or more of public hospitals in the country. During dengue outbreaks, the reporting is daily rather than weekly. These daily or weekly data are collated by focal persons of the VBDC team with data being transferred upwards from township to district to state/region and finally to the Central VBDC Programme at the Department of Public Health. The Central Programme also reports weekly to the Ministry of Health and Sports. From the Ministry of Health and Sports, the reports are sent to WHO SEARO through the WHO Country Office of Myanmar.

##### Dengue vector control measures

When a new dengue patient has been identified in an urban ward, the following measures take place within a 100-m radius from the patient’s house within 1 week of diagnosis: (i) larval control: insecticide granules (Temephos) are placed into domestic and peri-domestic water containers every 3 months in the wet season to kill mosquito larvae; (ii) mosquito control: space spraying (thermal fogging) using malathion insecticide; and (iii) health education from the VBDC teams by conducting health education sessions, distributing pamphlets, posters, and vinyl printed materials to the general public and transmitting information through mass media channels.

##### Dengue serotypes

During an outbreak of dengue, the National Health Laboratory requests hospitals at selected regions such as Yangon, Mandalay, Sagaing, Nay Pyi Taw, Mon, and Tanintharyi to send specimens from patients with severe complications. Serology was performed assessing for dengue viral-specific antibodies using the PAMBIO™ Dengue duo IgM and IgG rapid strip tests. Conventional PCR was then carried out on samples testing seropositive to determine the different serotypes according to the laboratory guidelines. This process is also carried out every year in children from the selected regions.

### Study sample and population

The study includes all patients diagnosed with dengue in public hospitals in Myanmar between 2011 and 2015. The study also includes information on dengue source reduction measures during the same time period as well as the pattern of dengue serotypes from patients residing in selected states/regions.

### Variables, data sources, and data collection

Data variables for the study included the following: For hospital-reported cases of dengue and deaths—year, month, age group, gender, urban/rural residence, disease severity grade, season, and ecological area of the country [16] for each case. The source of data was the VBDC electronic EXCEL database for aggregate data. For dengue vector control measures: (i) larval control—year, state/region, and coverage in townships, wards, households, and water containers; (ii) space spraying—year, state/region, and coverage in townships, wards, households, and schools; and (iii) health education—year, number of health education sessions, and number of people attending health education sessions. The source of data was the VBDC electronic EXCEL database for aggregate data from selected states/regions. For dengue serotypes—year, samples tested, samples seropositive, pattern of serotypes (1, 2, 3, or 4). The source of data was the National Health Laboratory electronic EXCEL database. The operational definitions for the study variables are explained in Table 1.

**Table 1:** Operational definitions of study variables

SN	Variables	Operational definitions
1	Ecological regions:	
1.1	Delta and lowland (heavy rainfall more than 2500 mm)	Ayeyarwady, Yangon, and Bago regions; Mon and Kayin states
1.2	Hills (moderate to heavy rainfall)	Kachin, Kayah, Chin, and Shan states
1.3	Coastal (heavy rainfall more than 2500 mm)	Rakhine state and Taninthayi regions
1.4	Plains (uneven topography and rainfall less than 1000 mm)	Magway, Mandalay, Sagaing, and Nay Pyi Taw regions
2	Disease severity grade: (WHO grading of severity of DHF)	
2.1	DF	Fever with two of the following: headache, retro-orbital pain, myalgia, bone pain, rash, and hemorrhagic manifestations (No evidence of plasma leakage)
2.2	DHF grade I	Fever and hemorrhagic manifestation (positive tourniquet test) and evidence of plasma leakage
2.3	DHF grade II	As in grade I plus spontaneous bleeding
2.4	DHF grade III	As in grade I or II plus circulatory failure (weak pulse, narrow pulse pressure (≤ 20 mmHg), hypotension, restlessness)
2.5	DHF grade IV	As in grade III plus profound shock with undetectable blood pressure and pulse

*WHO* World Health Organization, *DF* dengue fever, *DHF* dengue hemorrhagic fever

### Analysis and statistics

Data were extracted and cleaned in the EXCEL File and then exported to EpiData software for analysis (version 2.2.2.183, EpiData Association, Odense, Denmark). A descriptive analysis was performed using frequencies and proportions. Absolute numbers of dengue cases and deaths per year were determined and case rates per 100,000 people and dengue case fatality rates per 1000 cases were calculated. Cases and deaths were also analyzed in relation to age group, gender, urban/rural residence, disease severity grade, season, and ecological area of the country. Nationwide spot maps of dengue cases and deaths across 5 years (2011–2015) by ecological regions were generated through a geographical information system using Quantum GIS software (version 2.18.3, Open source Geospatial Foundation Project).

## Results

### Hospital-reported cases of dengue and dengue deaths

**Fig. 1:**
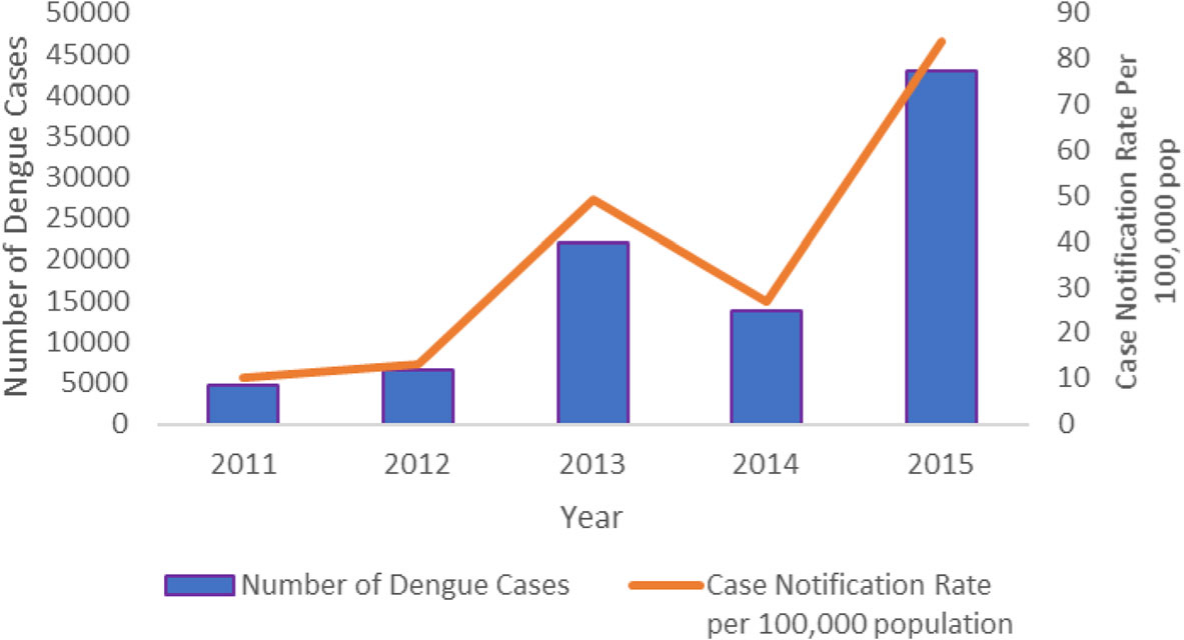
Annual hospital-reported cases of dengue in Myanmar between 2011 and 2015

**Fig. 2:**
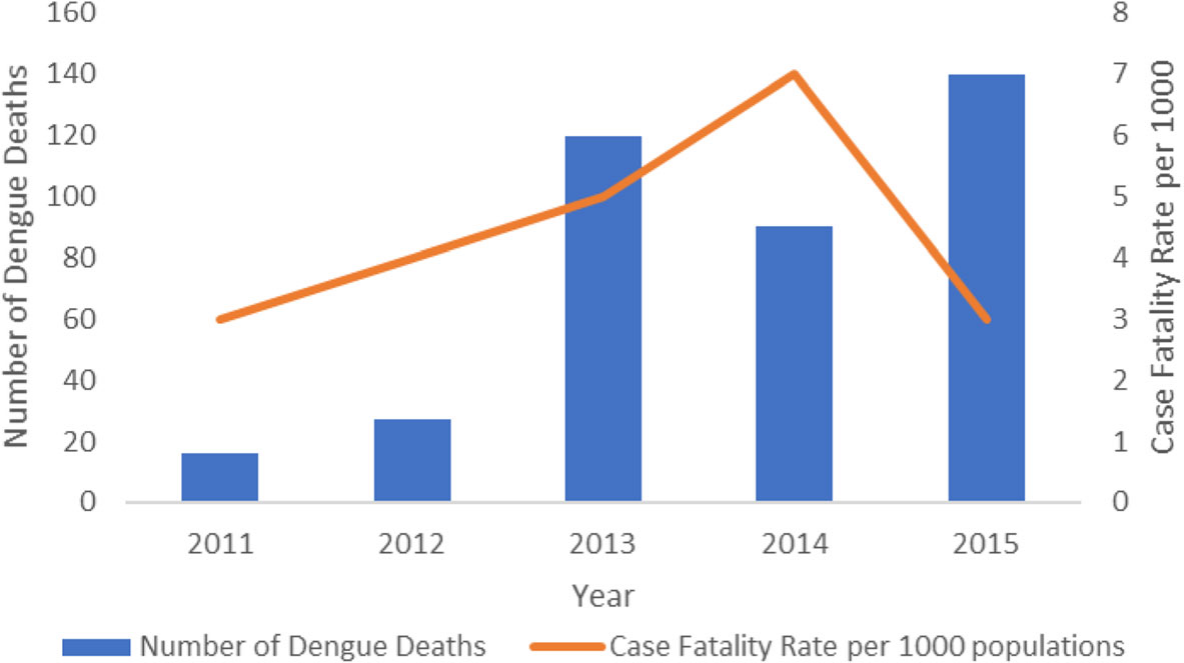
Annual hospital-reported dengue deaths in Myanmar between 2011 and 2015

The annual number of hospital-reported cases of dengue and associated deaths are shown in Figs. 1 and 2. During the five-year period, there were a total of 89,832 cases and 393 deaths. In 2013 and 2015, there was an increase in number of cases at 21,942 and 42,913, respectively, while during the other 3 years the number of cases ranged from 4738 to 13,806. The distribution of dengue deaths each year mirrored the distribution of cases. The case fatality rate was highest in 2014 at 7 per 1000 dengue cases, while in the other years, this ranged between 3 and 5 per 1000 cases.

The demographic and clinical characteristics of all the hospital-related dengue cases and deaths between 2011 and 2015 are shown in Table 2. Over 95% of all cases were in children, with those aged 5–9 years being the predominant age group affected. High case fatality rates were observed in infants < 1 year and adults ≥ 15 years. No differences were found in cases or deaths between males and females or those residing in urban versus rural settings. The commonest disease severity grade was DF plus DHF grade 1 (42%), although the majority of deaths and the highest case fatality occurred in those with disease severity grade IV.

**Table 2:** Demographic and clinical characteristics in relation to all hospital-reported dengue cases and dengue deaths in Myanmar between 2011 and 2015

Characteristics	Dengue cases		Dengue deaths		Case fatality per 1000
	*n*	(%)	*n*	(%)	
Total	89,832	(100)	393	(100)	4
Age group in years					
< 1	5049	(6)	42	(11)	8
1–4	22,613	(25)	100	(25)	4
5–9	42,186	(47)	187	(48)	4
10–14	17,146	(19)	45	(11)	3
≥ 15	2838	(3)	19	(5)	7
Gender					
Male	43,875	(49)	180	(46)	4
Female	45,957	(51)	213	(54)	5
Residence					
Urban	43,282	(48)	194	(49)	5
Rural	46,550	(52)	199	(51)	4
Disease severity grade					
DF and DHF grade I	37,771	(42)	16	(4)	0
DHF grade II	19,244	(21)	20	(5)	1
DHF grade III	14,285	(16)	44	(11)	3
DHF grade IV	18,532	(21)	313	(80)	17

*DF* dengue fever, *DHF* dengue hemorrhagic fever

**Fig. 3:**
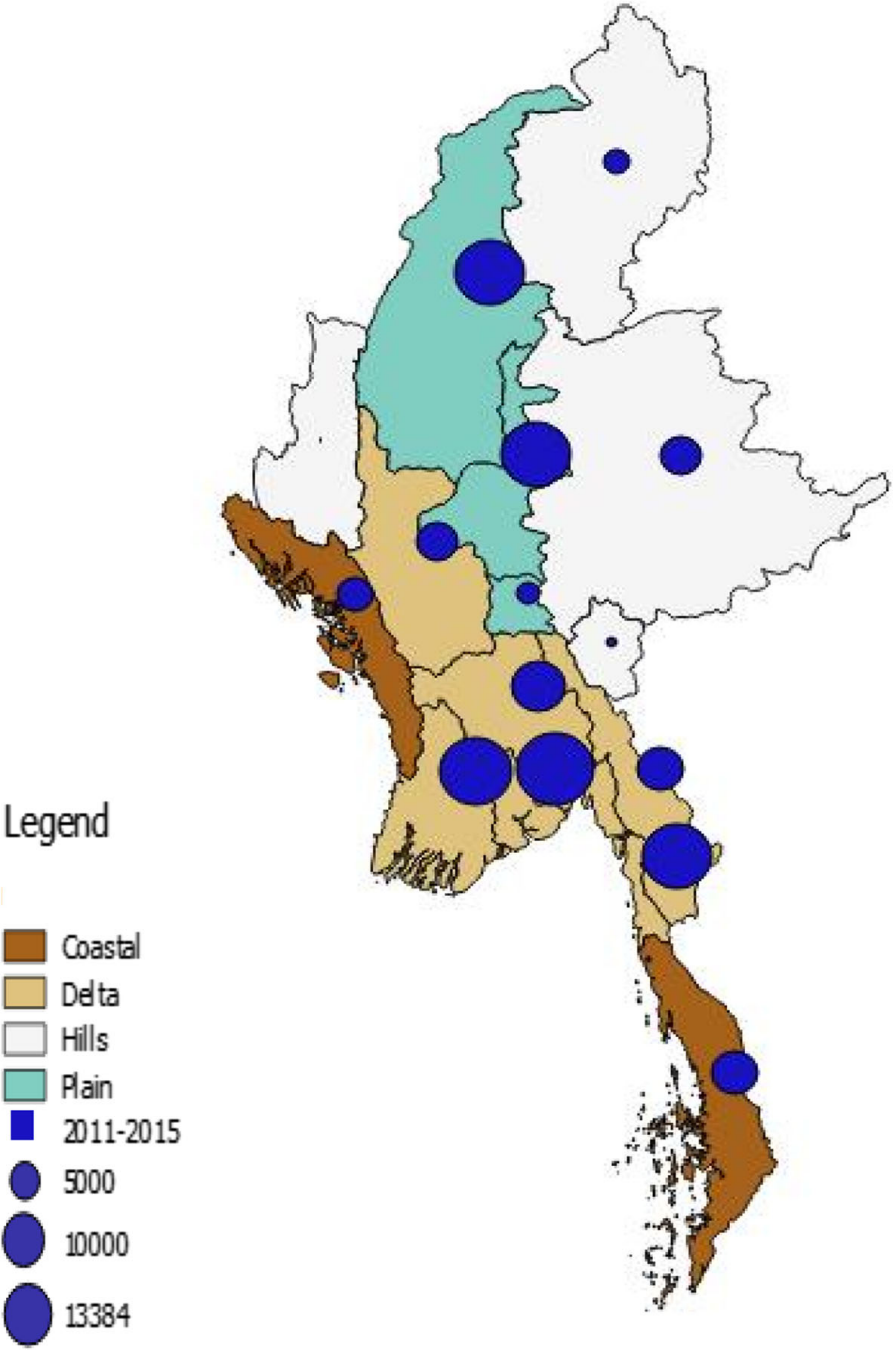
Distribution of all hospital-reported dengue cases in Myanmar between 2011 and 2015

**Fig. 4:**
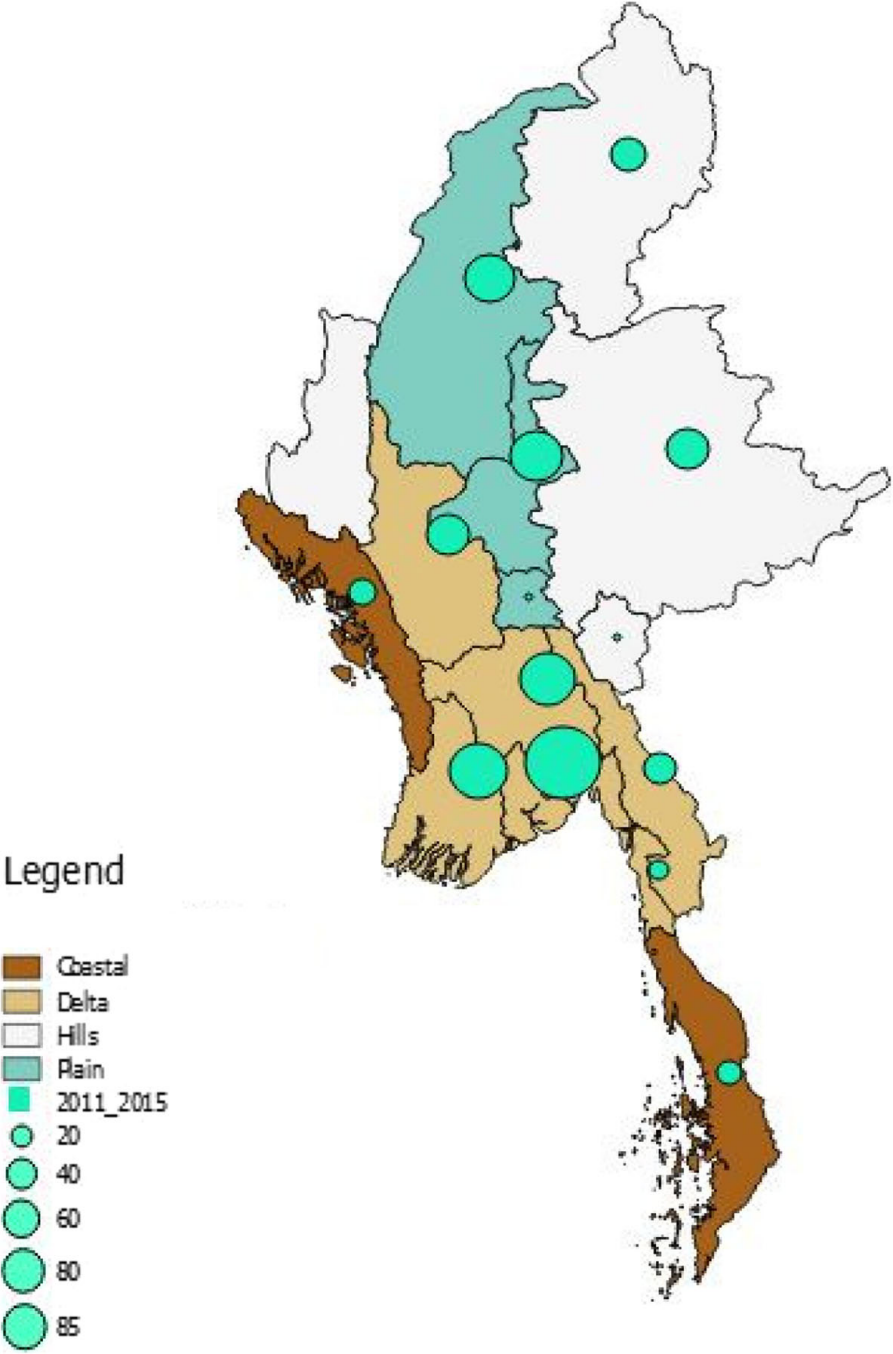
Distribution of all hospital-reported dengue deaths in Myanmar between 2011 and 2015

The seasonal changes and ecological distribution of hospital-related dengue cases and deaths between 2011 and 2015 are shown in Table 3. The majority of cases and deaths, with the highest case fatality rates, occurred in the wet season from May to September. The majority of cases (85%) and deaths (82%) also occurred in persons living in the delta region and the plains, although case fatality was highest in the hills. Figs. 3 and 4 show dengue cases and deaths in relation to the four ecological regions of the country. The five main hot spots for cases were Mon state and the four regions of Yangon, Ayeyarwady, Mandalay, and Sagaing, and the main hot spots for death were Yangon followed by Ayeyarwady region.

**Table 3:** Seasonal changes and the ecological distribution of hospital-reported dengue cases and dengue deaths in Myanmar between 2011 and 2015

Characteristics	Dengue cases		Dengue deaths		Case fatality per 1000
	*n*	(%)	*n*	(%)	
Total	89,832	(100)	393	(100)	4
Season:					
Hot (February to April)	4424	(5)	12	(3)	3
Wet (May to September)	75,171	(84)	330	(84)	4
Cool (October to January)	10,237	(11)	51	(13)	1
Ecological region:					
Delta and lowland	48,412	(54)	205	(52)	4
Hills	5732	(6)	49	(12)	9
Coastal	8327	(9)	24	(6)	3
Plains	27,361	(31)	115	(30)	4

### Dengue vector control measures and health education

The implementation of dengue vector control measures in townships, wards, households, and water containers is shown in Table 4. The implementation of larval control measures varied from year to year, although coverage progressively increased at township, ward, and household levels between 2013 and 2015 (Table 4). The proportion of water containers for which there were appropriate larval control measures was low at < 10% coverage in the last 2 years. The implementation of space spraying as a chemical control measure, especially during outbreaks progressively increased in townships and wards between 2012 and 2015, although coverage at the household and school levels was low, often at < 1% (Table 4). Health education sessions for the general public on dengue prevention and control along with numbers attending each year are shown in Table 5, the main findings being a large increase in education sessions and persons attending these sessions, particularly in 2014 and 2015.

**Table 4:** Annual implementation of dengue vector control measures in Myanmar between 2011 and 2015

	Total number^a^	2011		2012		2013		2014		2015	
		*n*	(%)	*n*	(%)	*n*	(%)	*n*	(%)	*n*	(%)
A: larval control measures											
Implementation of larval control measures at:											
Townships	330	277	(84)	81	(25)	68	(21)	157	(48)	152	(46)
Wards	3065	2655	(87)	931	(30)	1364	(45)	1716	(56)	2026	(66)
Households	10,889,348	2,753,002	(25)	2,191,259	(20)	1,790,151	(16)	2,729,804	(25)	3,447,524	(32)
Water containers^b^	51,400,000	29,666,676	(58)	5,420,553	(11)	6,755,019	(13)	4,497,076	(9)	10,954,867	(21)
B: space spraying measures											
Implementation of space spraying measures at:											
Townships	330	120	(36)	15	(5)	68	(21)	84	(25)	158	(48)
Wards	3065	855	(28)	28	(< 1)	478	(16)	1206	(39)	2026	(66)
Households	10,889,348	31,455	(< 1)	39,169	(< 1)	26,293	(< 1)	43,744	(< 1)	410,117	(< 1)
Schools	41,000	305	(< 1)	441	(1)	139	(< 1)	1797	(4)	2568	(6)

^a^Total number = total number in the country and is the denominator against which the percentages are calculated

^b^The estimated total number of water containers in Myanmar was based on one container per person living in Myanmar. Previous unpublished surveys have suggested that each resident in the country has one or slightly more than one container with the types of container being drums, cement tanks, ceramic jars, flower vases, car tires, tins, and coconut shells

**Table 5:** Annual number of health education sessions about dengue control measures and numbers attending these sessions in Myanmar between 2011 and 2015

	2011	2012	2013	2014	2015
	*n*	*n*	*n*	*n*	*n*
Health education sessions	17,928	16,139	6005	72,045	93,664
Persons attending health education sessions	2,608,181	1,793,324	2,254,388	3,137,995	4,841,742

### Dengue serotype patterns

Dengue serotype patterns between 2013 and 2015 are shown in Table 6. In the 2013 outbreak, serotype 1 predominated, while in the 2015 outbreak, serotypes 1, 2, and 4 predominated.

**Table 6:** Dengue serotypes identified each year in Myanmar between 2011 and 2015

Dengue serum samples and serotypes	2013		2014		2015	
	*n*	(%)^a^	*n*	(%)^a^	*n*	(%)^a^
Total serum samples tested:	230		21		37	
Samples seronegative	148		20		1	
Samples seropositive:	82		1		36	
Serotype 1	44	(54)	–		9	(25)
Serotype 2	4	(5)	–		11	(31)
Serotype 3	11	(13)	1	(100)	4	(11)
Serotype 4	23	(28)	–		12	(33)

^a^Numbers in percentages are column percentages and refer to serum samples seropositive

## Discussion

This is the first published study from Myanmar assessing the national burden and characteristics of hospital-reported dengue cases and deaths over a 5-year period, describing dengue vector control measures and health education in the community and reporting national health laboratory data on serotypes. There were some interesting findings.

The burden of hospital-recorded cases was high and mostly concentrated in children. In two of the five years (2013 and 2015), there were dengue outbreaks, which are variably defined, but in this context equate to case numbers that were significantly increased compared with the previous 5 years [2]. The annual pattern of dengue deaths mirrored that of cases, although, interestingly, case fatality was highest in the 2014, the year between the two outbreaks. The reasons for this are not completely clear, but may relate to the national census being carried out in 2014 providing more accurate population data and a shift to dengue virus serotypes 2 and 4 (which were more common in the 2015 outbreak) and which may be linked to secondary infections and more severe disease [17].

With the 5-year data combined, the highest case fatality was observed in infants and adults (aged 15 years and above). Unfortunately, in the adult population, the national information systems only capture those aged 15 years and above with no further stratification, so we do not know which of the older age groups are mainly at risk. A recent systematic review highlighted the importance of old age and non-communicable diseases, such as diabetes mellitus, respiratory disease, and renal failure as risk factors for severe forms of dengue [18], so older adults may particularly be susceptible to DHF and DSS.

Not surprisingly, disease grade 4 accounted for the majority of deaths and was associated with the highest case fatality. The vascular permeability, thrombocytopenia, liver pathology, complement activation, and altered hemostasis in severe dengue have been well characterized through autopsy studies on children dying of dengue in Myanmar [19], but to date, treatment is still supportive effectively with no specific antiviral agents available for targeted therapy.

The finding that dengue cases were more common in the wet season and in the delta and lowland regions concurs with current knowledge about seasonal transmission. High temperatures, relative humidity, heavy rainfall in the wet season, and poor drainage not only assist the main vector, *Aedes aegypti*, to multiply and adapt to its urban environment, but these factors also favor the propagation of dengue viruses in the mosquito itself [20–23] Case fatality was higher in the hills, and this may relate to health facilities being not as well equipped in these areas, health staff less experienced in the diagnosis and management of dengue, and poor immunity of these populations to the dengue virus [2].

It was encouraging to see the progressive increase in implementation of larval control measures and space spraying in townships and wards, particularly from 2013 to 2015, and this was accompanied by a similar increase in dengue health education sessions and the number of people who attended these sessions. Why there was a decrease in vector control measures between 2011 and 2012 is not clear, but may have been due to poor community participation and engagement at this time. Despite the increases in vector control measures in the most recent years, the implementation coverage of larval control measures in households and for water containers that reflected community participation was still low at less than 25% and space spraying at households and at schools was even lower at less than 10%. We do not know whether this reflects the true picture or is an indicator of weak monitoring systems, but clearly, the intensive vector control efforts over the last few years did not prevent the dengue outbreak of 2015.

Finally, the serotype data show that dengue serotype 1 predominated in the 2013 outbreak, with all four serotypes participating in the 2015 outbreak. These findings accord with previous published studies confirming the predominance of serotype 1 and the lack of correlation between serotypes and clinical severity in the 2013 outbreak [24] and the circulation of all four serotypes in the 2015 outbreak [25].

The strengths of this study are the large data set of hospital-recorded cases and deaths from 90% or more of public hospitals in the country and dengue source control measures from selected states/regions, which make the findings representative of what is happening in the country. The conduct and reporting of this observational study are also in line with internationally recommended STROBE guidelines [26]. However, there are a number of limitations. First, the cases and deaths were all health facility based, we do not know from the records whether the dengue cases were based on admission or discharge diagnoses, and there is no information in our study about the community burden of dengue or about the burden of asymptomatic infections. Lack of information on asymptomatic infections is an important epidemiological gap as a recent study has shown that people with asymptomatic infections not only infect mosquitoes, but are more infectious to mosquitoes than those with dengue symptoms [27]. Second, we were unable in dengue cases classified as grade I to differentiate between DF and DHF. Third, we do not know (as stated earlier) whether the low implementation coverage of larval control or space spraying measures in households and schools or in water containers is a true reflection of what is happening or whether this relates to weak monitoring, recording, and reporting. Finally, the data used in the study were all secondary and collected through the routine systems, so we have no means of checking accuracy or validity.

Despite these limitations, there are three important programmatic implications from this study. First, the health facility-based reporting of dengue cases and deaths needs to be revisited to ensure there is functionality at full capacity, the data are collected and transmitted in a timely way, and through regular supervision, the data are quality assured. It is also important for the program to collect a more detailed age-breakdown for those aged 15 years and above and to divide disease severity grade I into DF and DHF. Whether monitoring can extend to community-level disease and asymptomatic infection needs further discussion, but more accurate measurements and estimates of disease burden will be essential for planning vaccination campaigns. Vaccination, in combination with vector control, is seen as a promising tool in the fight against dengue. Currently, there are three candidate live-attenuated vaccines in phase 2/3 (NIH TV003/TV005 and Takeda TDV vaccines) or phase 4 (Sanofi Dengvaxia) trials, although there are many challenges to overcome before efficacy and safety are assured [28].

Second, there is need to strengthen (a) the monitoring and (b) recording of vector control measures, particularly for households, schools, and water containers. Vector control represents a substantial portion of government dengue-related costs, and it is important to note that control of *Aedes aegypti* is likely to impact not only on dengue but also on chikungunya and *Zika* viruses. The chikungunya virus has been detected in Myanmar [29, 30], but due to overlap in the clinical presentation with dengue and cross-reactivity of the serological tests, the burden of disease in the country has not been determined. *Zika* virus was first detected in a foreign national in Myanmar in 2016 [31], but to date, there are no published reports of the burden of *Zika* virus in the country.

Third, basic science research is crucial to understand more about the virus (serotypes, genotypes, and strains), the immune responses, the immune correlates of protection and risk, and the development of dengue vaccines. National programs, however, would do well to work in partnership with non-governmental organizations, local community-based organizations, and self-help groups to ensure better case management, develop and use point-of-care diagnostics, undertake timely and accurate surveillance/monitoring and reporting, to better engage with the community, to respond to outbreaks, and to implement source reduction measures in households as well as in public spaces [28, 32, 33]. More attention also needs to be given to novel biological, genetic, and behavioral approaches that target mosquitoes such as the use of Wolbachia bacteria strains that infect *Aedes aegypti* mosquitoes [34] and genetically modified mosquitoes carrying lethal genes [35].

## Conclusions

In conclusion, this study has described the large burden of hospital-recorded dengue in Myanmar between 2011 and 2015, which was concentrated predominately in children and which included two outbreaks in 2013 and 2015. Case fatality was particularly high in infants aged less than 1 year, adults aged 15 years or more, patients with grade IV disease, and those residing in hilly regions of the country. Implementation and coverage of dengue vector control measures, including larval control, space spraying, and health education, all increased between 2012 and 2015, although there are concerns about low coverage of these interventions in households and schools. Dengue virus serotype 1 predominated in the 2013 outbreak, while all four serotypes circulated in the 2015 outbreak. Dengue is a serious public health disease burden in Myanmar. More attention is needed to improve the monitoring, recording, and reporting of cases, deaths, and vector control activities, and to avoid misclassification bias in reporting hospitalized dengue cases, the notification systems must just report the discharge diagnosis. Finally, more investment needed for programmatic research.

## Abbreviations

DF/DHF: Dengue Fever/Dengue Hemorrhagic Fever
DSS: Dengue shock syndrome
VBDC: Vector-borne disease control
WHO: World Health Organization
